# Description of the Australian pork supply chain and implications for national biosecurity management

**DOI:** 10.1111/avj.70011

**Published:** 2025-09-13

**Authors:** P Schrobback, J Aboah, K Richards, R van Barneveld, S McFallan, J Langbridge

**Affiliations:** ^1^ Commonwealth Scientific and Industrial Research Organization (CSIRO), Agriculture and Food St Lucia Brisbane Australia; ^2^ WorldFish, Inclusive Aquatic Food Systems Nairobi Kenya; ^3^ SunPork Group Eagle Farm Brisbane Australia; ^4^ Commonwealth Scientific and Industrial Research Organization (CSIRO) Dutton Park Queensland Australia; ^5^ Teys Australia Limited Eight Mile Plains Brisbane Australia

**Keywords:** Australia, control measures, disease, disruptions, material flow, pork, supply chain

## Abstract

Outbreaks of emergency animal diseases such as African swine fever (ASF) and foot‐and‐mouth disease (FMD) are typically managed through regulated control measures, including tracing, surveillance, movement restrictions, culling, disposal and decontamination. However, limited understanding and fragmented data on material flows – such as semen, live animals and meat products – within meat supply chains hinder policymakers' ability to assess the full impact of these measures and to consider these implications in their decision‐making. This study aimed to map the material flow within the Australian pork supply chain and to identify the potential socio‐economic implications of disease control interventions. Industry experts were engaged to assist in the drafting of a flow chart and to provide descriptions of activities at each segment of the supply chain. Results revealed a highly integrated and complex supply network. These structural and operational features, combined with regulatory movement controls, can lead to significant disruptions, including loss of livestock and breeding capacity, business income and employment, animal welfare risks, psychological stress, reputational damage from mass culling and reduced meat availability for consumers. The findings of this work emphasise the importance of decision‐makers being well informed about the effects of supply chain disruptions and the socio‐economic consequences of disease control decisions.

**Abbreviations**
AADIS: Australian animal disease spread
ASF: African swine fever
CO_2_
: Carbon dioxide
DC: Distribution (accumulation) Centre
EAD: emergency animal disease
EADRA: emergency animal disease response agreement
FMD: food‐and‐month disease

The continuous and safe supply of meat products to consumers is important for food and nutritional security, given the high value of animal proteins in human diets.^1^ Emergency animal disease (EAD) outbreaks and responses to such events, among other disruptions, can significantly affect animal protein supply for human consumption. EADs can also affect the resilience of livestock industries. The literature offers a broad range of studies that assess the impacts of EADs on society.^2^, ^3^, ^4^, ^5^, ^6^ For example, livestock industry stakeholders may be affected in various ways including private and public costs of controlling the disease, income losses for producers due to animal mortality/morbidity, human health impacts due to zoonoses, reduced animal/meat availability, increased meat prices affecting affordability, loss of access to export markets, compromised animal welfare and loss of consumer confidence.^2^, ^3^, ^4^

However, previous studies did not specifically focus on the implications of disease control measures which governments typically implement to minimise the risk of EAD spread, for example, biosecurity, tracing, surveillance, movement controls, destruction, disposal, decontamination, quarantine and export/import restrictions.^7^ Furthermore, existing studies have not applied a supply chain perspective to the socio‐economic implications of disease control measures. Such nuanced information can benefit government authorities in better understanding the type and scale of implications and impacts that disease control measures may carry, allowing them to consider these against the type, spatial and temporal extent of controls. These are the knowledge gaps that this study aimed to address using the pork supply chain in Australia.

Pork meat is the second most consumed animal‐based protein (20.6 kg/capita) after poultry (31.5 kg/capita) in Australia.^8^, ^9^ The Australian pork industry is currently free of EADs such as African swine fever (ASF) and foot‐and‐mouth disease (FMD). Pre‐ and postborder measures are in place to prevent, prepare and respond to EAD outbreaks, for example, the Emergency Animal Disease Response Agreement (EADRA) and AUSVETPLAN.^10^ Disease preparedness planning also includes an economic benefit assessment of targeted EAD response strategies.^11^ However, due to the limited understanding and fragmentation of information about the distribution network of pork meat produced in Australia, industry representatives and government authorities in Queensland and New South Wales identified the need for government authorities to better understand the potential socio‐economic implications of regulated restrictions on the material flow within the pork supply chain due to EAD events. Such information is considered the foundation for more informed regulatory decision‐making and for further improving the preparedness of supply chain stakeholders to respond to potential future EAD events.

Therefore, this study aimed to (1) map and describe the material flow within the Australian pork supply chain and (2) identify the potential socio‐economic implications of regulatory controls on this material flow in the context of an EAD outbreak.

## Methods and information sources

The focus of this study was on the implications of regulated controls on the flow of material in the meat supply chain, for example, animal semen, live animals, primary meat products, coproducts, by‐products, the destruction (culling) of animals and the closure or disruption of abattoirs.

The study adopted a qualitative assessment approach to gain in‐depth information about stakeholders and processes within the material flow network and the breadth of potential socio‐economic implications due to regulated movement controls. The specific methods employed under this research approach included a scoping literature review, a stakeholder workshop, stakeholder interviews and one reconnaissance tour of a pork abattoir.

A scoping literature review was conducted as an initial step to determine the breadth of the existing literature on the Australian pork supply chain (e.g., stakeholder groups, links, processes). The literature search included journal articles and grey literature (e.g., industry and government reports). The databases Google Scholar and Science Direct were used for the search of keywords and abstracts. The following terms and Boolean search strings were used: ‘pork’ OR ‘pig’ AND ‘supply chain’ OR ‘movement’ AND ‘Australia’. The search resulted in 17 publications that included relevant pork supply chain aspects.^12^, ^13^, ^14^, ^15^, ^16^ None of these publications offered a comprehensive structure with an in‐depth description of stakeholders and processes within the supply chain.

The information from previous publications was summarised in the form of a preliminary pork supply chain map. Identified gaps within the preliminary supply chain map (e.g., need for more detailed information of specific processes in upstream and midstream segments, potentially missing links and stakeholders) were recorded in the form of questions that were to be explored in further detail in a stakeholder workshop and stakeholder interviews. The questionnaire also included the question about the potential socio‐economic impacts of EADs on individual supply chain stakeholders (e.g., producers and abattoirs) due to regulated movement controls on material flows. The engagement with supply chain stakeholders followed a semistructured workshop/interview format, allowing the research team flexibility and adaptability to explore new information in depth (e.g., uncover unexpected information, clarify reasoning).

A pork supply chain stakeholder workshop was conducted in August 2023. Participation in this workshop included the fulfillment of at least one of the following recruitment criteria: (1) is a stakeholder in the pork supply chain (e.g., producer, logistics provider), (2) is a representative of the pork/livestock industry association, (3) is an agriculture/livestock policymaker and (4) is a veterinarian or biosecurity expert. Additionally, two of the authors contributed valuable operational insights in their capacity as senior executives from a mainstream pork supply chain business. The workshop was held in a hybrid form allowing in‐person and online participation. Workshop participants were first introduced by the research team to the project context. Participants contributed to the development of a detailed pork supply chain map and description of activities included in the map. In the final step, the potential socio‐economic impacts of EADs on individual supply chain stakeholders were discussed.

The same procedure and participant recruitment criteria were used for stakeholder interviews including with participants who were unable to attend the workshop and additional stakeholders to gather further insights, specifically on matters that remained unclear after the workshop. The interviews were conducted in person or online and took about 1 h to complete. The interviews were conducted between October 2023 and April 2024. In total, 37 participants were engaged in primary data collection (see Table 1).

**Table 1:** Overview of workshop and interview participants

Stakeholder group	Number of workshop participants	Number of interview participants	Total number of participants
Boar stud operator	3	2	5
Producer	5	‐	5
Meat processor	6	2	8
Renderer	‐	1	1
Retailer	‐	‐	‐
Livestock transport provider	2	2	4
Industry peak body^a^	2	1	3
Government authority	9	1	10
Consultant	1	‐	1
Total	28	9	37

^a^ Includes peak bodies for producers and livestock transporters.

The workshop and interviews were audio‐recorded and transcribed using Microsoft Teams for data analysis purposes after participants provided their consent in either written (workshop) or verbal (interviews) form. A word processing approach using Microsoft applications was employed to analyse data (e.g., links and processes within the supply chain, themes surrounding social‐economic impacts of potential EADs). The focus was on identifying the type and breadth of potential impacts from material movement controls on affected stakeholders. A recording of frequencies by which each individual impact was mentioned during workshop and interviews would not have been meaningful since some participants were more familiar with their segment of the supply chain than with others (e.g., logistics providers vs retailers vs government authorities).

The primary data collection process also involved a reconnaissance tour of one export‐certified pork abattoir in Queensland. The tour aimed to provide the research team with an understanding of animal and meat handling processes during the processing stage of the supply chain as well as the identification of stakeholders who obtain material from abattoirs. Data collected from the tour included written notes on observations and comments that the tour guide provided. The data were analysed in the same manner as information collected from the workshop and interviews. Overall, primary data collection was continued until no new information occurred and all remaining questions were answered.

### Animal ethics statement

Ethics and privacy approval for the primary data collection was obtained from the first author's organizational Social Science Human Research Ethics Committee and Privacy Committee under approval number 105/23. The approval was provided in August 2023.

## Key findings

This section presents the map and a brief description of the derived material flow within the Australian pork supply chain. This section also includes results from the theme analysis of potential socio‐economic implications of regulated movement controls, animal destruction and abattoir closure on the flow of material in the Australian pork supply chain due to an EAD event. Additional results are provided in the S1.

### Material flow within the pork supply chain

**Figure 1:**
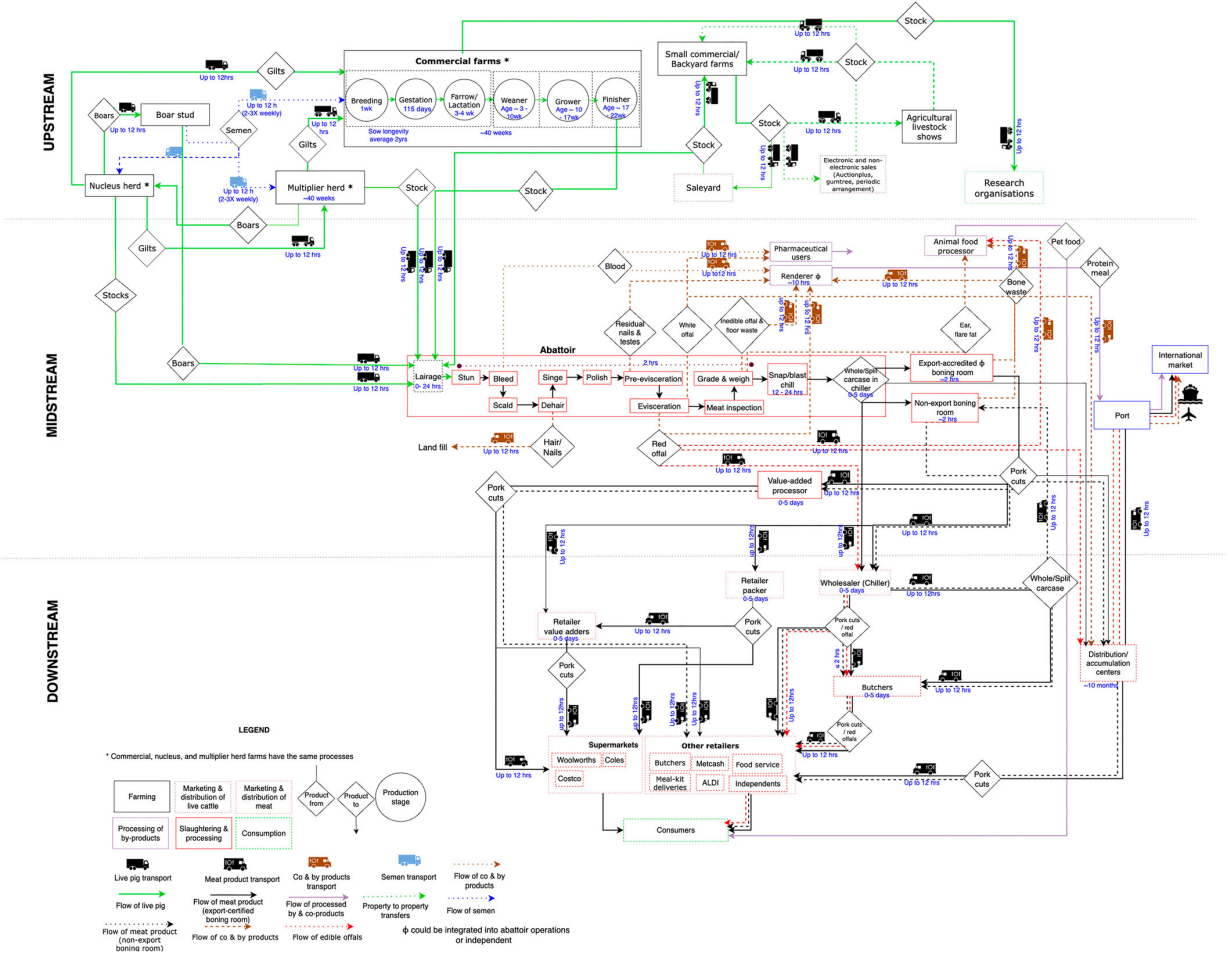
Material flow and approximate timelines within the Australian pork supply chain.

The material flow within the pork supply chain (Figure 1) starts with semen supply from boar studs. Over 90% of commercial pig breeding relies on artificial insemination using domestically produced, fresh semen from boar studs.^10^ Only a small proportion of pig farms, mostly small commercial, smallholder and pig‐keeper farms, use natural mating as a reproduction method. Fresh pig semen is delivered from boar studs to nucleus herds, multiplier herds and commercial farms two to three times a week. Nucleus herds are breeding herds used to produce genetically superior pigs and are typically closed breeding systems, that is., no external pig intake. Boars from the nucleus herds are sent to the boar studs for semen collection. Multiplier herds source their gilts (female pigs that have not yet bred) from a nucleus herd and semen from a boar stud to produce gilts for commercial pig farms.

At commercial farms, groups of sows are artificially inseminated each week, resulting in pigs being born (farrowed) in weekly cycles. This process is known as continuous farrowing. The subsequent lifecycle stages include lactation, weaning, growing and finishing. The process from breeding the sow to finishing the progeny in a commercial pig farm system takes about 40 weeks.

Commercially grown pigs are typically transported (via internal and external live animal logistics providers) up to three times in their lifetime, depending on the type of production system. Commercial farms also occasionally provide pigs to universities and other research organisations for research and development purposes.

Activities at small commercial/smallholder/pig‐keeper farms include breeding, farrowing, lactation, weaning, grow‐out and finishing of pigs. For breeding at small commercial/smallholder/pig‐keeper farms, boars may be moved from and to different farms for mating (periodic arrangements).

The majority of pigs nationally are sold directly for processing at a specified abattoir. A very small number of pigs are sold through saleyards and mostly by smallholder/pig‐keepers. These pig producers are increasingly using online platforms like Gumtree (https://www.gumtree.com.au/s-livestock/c18457) and Farm Tender (https://www.australiantenders.com.au/search/tenders/) to sell pigs, with most sales on these platforms being unregulated and lacking prior veterinary assessment. Pigs are a popular feature at some agricultural shows in Australia. For these shows, pigs are typically moved from small commercial/smallholder/pig‐keeper farms to the location where the agricultural shows are taking place (mostly intrastate movements).

Live animal logistics providers are trucking companies that transport live pigs from one point in the upstream supply chain to the next; though some pig producers transport their own pigs. All property‐to‐property and property‐to‐abattoir movements of live pigs are required to be recorded by dispatchers, transporters and receivers and reported to the PigPass (https://pigpass.australianpork.com.au/faq) database, which underpins the national pig traceability system (see Supplementary material).

The midstream segment of the supply chain consists of abattoirs, cold stores and ports. Australia currently has seven export‐certified pork abattoirs that process about 90% of pigs nationally and supply both domestic and export markets. Most of these abattoirs are part of an integrated supply chain model and include breeding, growing and processing of pigs within their business operations. In addition, there are five domestic abattoirs that collectively process about 10% of pigs produced in Australia.

At the export‐certified abattoirs, pigs arrive by truck and are off‐loaded in groups into lairage pens. In lairage (2–24 h at export‐certified pork abattoirs), pigs undergo an ante mortem health inspection by an Australian government veterinarian, with most receiving clearance for unconditional slaughter and proceeding to normal processing.

All seven of Australia's export‐accredited pig abattoirs use carbon dioxide (CO_2_) stunning.^17^, ^18^ In NSW, a high proportion of pigs (97%) across multiple pork processing facilities are stunned in this manner, while the remaining pigs processed at other, smaller domestic abattoirs are stunned via captive bolt or electrical stunning.^18^ Progeny pigs are usually stunned in small groups in a CO_2_ stunning chamber.^17^

After stunning, the pigs are bled by severance of the major neck vessels, and the carcases are scalded, dehaired, singed and polished. The skins are not removed from the carcases. Prior to evisceration, by‐products like nails and blood are removed and sent to a renderer. The evisceration process involves separation of red and white offal from the polished carcases. Red offal includes organs like the heart, liver, kidneys and tongue. White offal includes organs such as the stomach, intestines, lungs and pancreas.

The processes from stunning to chilling of the carcase take approximately 45 min (regulated maximum 2 h). Carcases are moved to a chilling room (snap or blast chill at around 4°C) and kept there for 12–24 h. From the chiller, carcases may be moved to either an export‐accredited boning room which is integrated into the abattoir facility, or offsite to an external export or nonexport‐certified boning room.

Whole or split carcases may also be supplied from an abattoir's chillers to wholesaler' chillers or cold stores. Products in cold stores may be exported (if processed at an export‐accredited abattoir), distributed to the domestic retail market or moved for further processing at a value‐added processor. Pork cuts leaving boning rooms are distributed to downstream supply chain entities including cold stores, wholesaler' chillers, value‐added (further) processors, retail value addition facilities or a retail packer. Red offal and white offal are cleaned, packed and transferred to cold stores where they are accumulated for export. Some red offal is distributed to the domestic market through wholesalers and butchers.

Some by‐products that are produced during processing, such as blood, require specialised transportation tanks, while other by‐products and coproducts are transported to renderers by large trailers or to pet food producers by refrigerated trucks.

Rendering is the process of managing by‐products from carcase processing, using heat to transform inedible offal as well as condemned carcases of injured/sick animals into secure, pathogen‐free feed proteins and other valuable by‐products.^19^ The high‐protein meals from rendering are used to produce pet and livestock food, for example, kibble, meat and bone meal, tallow (rendered fats) and oils and oleochemicals, for example, cosmetics, candles, soaps. Australian protein meal is also exported.

Pet food processors use by‐products and coproducts from animal processing at abattoirs and from renderers to produce wet or dry pet food for both export and domestic markets.

Downstream stakeholders that are involved in the further processing of meat products and the distribution of the products include value‐added processors, retail value packers, wholesalers, butchers, distribution accumulation centres (also known as DCs), supermarkets and other retailers. Domestic consumers purchase meat products or coproducts from retailers, for example, supermarkets, butchers or other retailers based on their food preferences. For home consumption, consumers or delivery services transport the meat products from the retail store to the consumer's home, where the meat products undergo their final processing and consumption or are discarded as waste (link not shown in Figure 1). A more detailed supply chain description is provided in the Supplementary material.

### Potential socio‐economic implications of mandated restrictions to material flow

The potential socio‐economic implications of regulatory controls on the material flow within the pork supply chain were derived from the data collected from the stakeholder workshop and interviews, with context provided by the material flow map. Key processes and stages within the supply chain that were identified as being exposed to the highest risks arising from disruptions due to movement control measures are (1) semen movement (upstream), (2) animal movement (upstream), (3) destruction of animals (upstream) and (4) closure of abattoirs or restrictions to outgoing movements from abattoirs (midstream). The identified theme descriptions of potential socio‐economic implications that movement control measures can have on these supply chain segments are summarised in Table 2 and explained in this section.

**Table 2:** Socio‐economic implications of regulatory control measures on the material flow in the Australian pork supply chain

Regulatory disease control measures (supply chain section)	Implications (theme descriptions)^a^	Affected key stakeholders
Restrictions on semen movement (upstream), for example, regulatory controls on semen movements or closure of boar studs	Supply shortages in the number of pigs that can be produced due to lack of semen (10 lost pigs per mating, or 500 pigs per week per 1000 sows at a loss of $4300 per sow or $215,000 weekly per 1000 sows, with ripple effect along the transport and processing chain occurring with a 40‐week time delay) Supply shortages in pork products (shortage of 800 kg of meat per lost mating, equating to 40 tonnes less meat per week per 1000 sows and associated ripple effects along the chain) (A) Financial loss for boar studs and producer genetic improvement programme lag Future loss of income for feed suppliers, transporters, abattoirs and downstream stakeholders due to pig shortages Future loss of income for pig breeders & producers due to the lag in pig breeding and grow‐out that cannot be made up later due to logistical, space and other factors Increased nonproductive sows and likelihood for sows to be prematurely culled due to lack of capacity to absorb nonmated sows into subsequent production weeks that align with their cycle, in combination with possible reduction in abattoir capacity to cull them. Delayed associated replacement costs to introduce new gilts several months later at the next cycle ($650/sow) Potential job/skill loss due to the reduced volume of material within in the chain (B) Psychological distress of all affected stakeholders (C)	Livestock feed providers (1), boar studs (2), pig producers (3), saleyards (4), livestock transporter, professional services (nutritionists, veterinarians) (5), abattoirs (6), exporters (7), secondary & value add processors (8), operators (9), wholesalers (10), logistics operators (11), retailers/food services (12), consumers (13), renderers (14), pet food processors (15), industry associations (16), government (17)
Restrictions on animal movement (upstream)	Disruption to regular, required pig movements including property‐to‐property for growing out, property‐to‐abattoir for slaughter and processing and property‐to‐property for incoming live replacement gilts, disruption to movements to/from small commercial/smallholder/pig‐keeper systems Longer holding times of animals on farms negatively affect animal welfare as new pigs are born and enter the system, but pigs are not exiting, for example, due to lack of sufficient space/housing, labour, feed, water and overstocking (D) Culling for animal welfare reasons (E) Pigs grow out of required specification for sale and continued growth compounds overstocking and welfare is compromised on farm Supply shortages in the number of pigs that can be processed (F) Financial loss for farms, for example, loss of income, destruction of animals, disposal of carcases Additional costs for farms, for example, feed and biosecurity compliance costs Financial losses for other operators in the supply chain including feed suppliers, transporters, abattoirs and downstream stakeholders (G) Negative public perception due to media coverage affecting the pig sector, public withdrawal of the social license to operate, also affecting government responders (H) Environmental implications of disposing carcases (J) A, B, C (see above)	1–17 (see above)
Destruction of animals (upstream)	A‐J (see above)	1–17 (see above)
Closure of abattoirs or disruption to outgoing movements from abattoirs (midstream)	A‐J (see above)	1–17 (see above)

^a^ Implications depend on the specific context, time and scope of disease control measures being implemented.

#### Implications of disruptions to semen supply (upstream)

Analysis of the collected data revealed a high reliance of commercial pig farms on regular, fresh semen supply from boar studs. Less than 0.1% of porcine semen product globally is cryopreserved, as porcine sperm are prone to cryoinjury.^20^ Similarly, commercially viable porcine embryo transfer processes do not yet exist. This implies that the closure of boar studs means that no sows can be inseminated for the period of closure. This creates a significant gap in pig production that cannot be recovered. Disruptions to the pig semen supply will create a ripple effect in the pork supply chain lasting 40 weeks and more (i.e., the period from insemination to finishing). The estimated lost income and additional costs incurred per lost mating – affecting upstream and midstream supply chain functions such as farms, boar studs, transporters and abattoirs – are approximately $4,300. For a medium‐sized herd of 500 sows, this equates to around $107,000 in losses per week. In terms of downstream impacts, it is estimated that each lost mating results in approximately 800 kg of meat not entering the supply chain, amounting to a weekly loss of about 20 tonnes for a 500‐sow herd (a detailed calculation is provided in the Supplementary material).

Downstream impacts include (1) widespread supply shortages in the number of pigs that are produced and subsequent pork products, (2) future loss of income for pig breeders and producers due to the lag in pig breeding and grow‐out, (3) increased need for sows to be culled as there is no capacity to absorb nonmated sows into subsequent production weeks that align with their cycle, that is, nonpregnant sows cannot be maintained in the herd for 20 weeks until their week batch is due to next be mated, (4) potential job/skill loss at pig farms, abattoirs and other downstream operators and logistics operators due to the reduced number of animals on farms/slaughtered at abattoirs and reduced incomes for farms/abattoirs.

Other stakeholder groups such as livestock feed providers, professional service providers, for example, nutritionists and veterinarians, government and industry associations will also be affected by these disruptions. It is estimated that 1 week of semen delivery disruption in Queensland alone, represented a loss of income and additional costs of approximately AUD 13 million to the Australian pig industry (Industry representative, April 2024).

#### Implications of disruptions to animal movements (upstream)

The supply chain map for pork meat (Figure 1) shows that there are several points within the network at which live animals are moved. For pigs in integrated commercial farming systems, the number of movements between different properties varies, but these movements generally occur within a state/territory boundary. Regulatory controls on pig movements will affect required, routine pig movements between properties for growing out and finishing pigs to abattoirs. These are considered to be the most important movements. Other typical transfers that will be affected by regulatory animal movement controls include relocation of live replacement gilts associated with nucleus and multiplier operations, transfers within small commercial/smallholder/pig‐keeper systems and to and from saleyards.

Study participants emphasised that in such situations, the farms where pigs are reared will need to hold the animals longer than in a business‐as‐usual case, which may rapidly cause significant challenges on farms. Commercial pig farms generally operate on a continuous production model, that is, weekly insemination, farrowing and finishing, with pigs usually housed indoors with limited housing space redundancy to accommodate new and existing pigs if outward pig movements are disrupted. Study participants highlighted that these operations will not have the capacity, including space, housing, labour, feed and water capacity, to hold pigs for more than 1–2 weeks in the case of disruption to regular movements. Overstocking due to such disruption will rapidly impact pig welfare and health, and if not rectified will lead to substantial on‐farm destruction of pigs for welfare reasons across many farms. Such measures can cause (1) financial and production loss for farms, (2) additional costs due to feed, destruction, disposal, biosecurity compliance and other requirements, (3) psychological distress to farmers/workforce/veterinarians, (4) negative public perception/reputation through media coverage affecting the entire pig sector and its social license to operate, also affecting government authorities and (5) environmental implications associated with disposal of pig carcases, among others.

Disruption of the movement of pigs will also affect livestock transport providers, livestock buyers (operators) and abattoirs since they cannot operate if pigs cannot move. This may cause layoffs/unemployment of skilled workers (e.g., truck drivers, meat boning staff) and significant financial loss or costs incurred for businesses (e.g., loss of income, compliance costs with biosecurity measures). These effects will ripple through to renderers, pet food processors and downstream supply chain stakeholders, for example, value‐added processors and wholesalers. Subsequently, the quantity of meat products available at the domestic retail and food service level and for potential export (if export markets remain) will decrease. It is likely that some/all of Australia's pork export markets will cease their import of Australian meat products in the event of an EAD outbreak. In the immediate short term only, this may offer an opportunity to divert meat products destined for export, to domestic markets. However, this is likely to only help to maintain food security in Australia in the short term. Impact due to pig movement controls will also impact service provider industries such as stockfeed and other companies.

#### Implications of destruction of animals for disease control (upstream)

Participants in the stakeholder workshop and interviews mentioned that any mandated destruction of animals at the farm level to control the spread of disease from high‐risk premises will result in fewer animals being available for slaughter, which will affect the quantity of meat available at the retail level and export (if export is permitted). Peripheral sectors such as renderers and pet food processors may also be affected by the decreased number of animals, as outlined in Implications of disruptions to animal movements (upstream) section. The extent of this impact will be determined by the number of pigs destroyed and potential implications, as outlined in Implications of disruptions to animal movements (upstream) section. Participants also noted that animal destructions due to welfare risk for animals under regulatory movement controls, for example, longer holding times, limited space/housing, would have similar, if not more severe implications (due to scale) compared with the destruction of animals for disease control.

#### Implications of regulated closures of abattoirs (midstream)

Midstream supply chain disruption such as the closure of an abattoir implies a halt to the flow of incoming live animals and outgoing meat. There is limited capacity to divert pigs to other abattoirs for reasons including that abattoir capacities to process additional animals are limited, animals may not be suitable (e.g., not Australian Pork Industry Quality Assurance accredited), transport may not be possible and movement controls may apply (e.g., cross border given small number of large abattoirs). Hence, negative pig welfare outcomes as described in Implications of disruptions to animal movements (upstream) section may occur. Longer interruptions at abattoirs also entail consequences for the workforce, for example, layoffs for a casual workforce. Depending on the length of the closure of an abattoir and the level of employee confidence, skilled/specialised labour may be lost. These skilled/specialised workers may seek other employment opportunities in other sectors and therefore may become unavailable if or when abattoir operations recommence. A shortage or loss of skilled/specialised labour would significantly affect the processing capacity of abattoirs upon recommencement, and their replacement may be associated with additional training and other costs. In addition, the abattoir businesses themselves would not be able to remain financially viable if they are not operating. Abattoir operations are optimised to a complex array of overhead, operating and compliance costs. Operating at as little as 15% below optimal capacity can be the difference between a sustainable and nonsustainable abattoir operation. Given that a small number of export‐accredited abattoirs process 90% of pigs in Australia, the loss of processing capacity due to business failure at any one of these abattoirs would reverberate across the industry, with lost processing capacity unable to be absorbed by other abattoirs.

Furthermore, within the midstream and downstream segments of the pork supply chains, there is limited capacity to store and process larger quantities of meat products; hence, abattoir closures could rapidly translate to meat shortages at the wholesale, food service and retail stages. For meat products that are stored, the shelf life for human consumption must be considered in managing their distribution during a period of shortage.

Hence, interruptions in any segment of the pork supply chain (semen, live animals or abattoir) will likely have immediate and escalating implications for supply chain stakeholder viability, pork meat availability at the retail level across Australia, depending on the specific context, time and scope of disruption. Due to a lack of meat supply, businesses within the downstream section of the meat supply chain may experience similar implications as businesses up‐ and midstream, including loss of income, jobs, skilled workers and psychological distress. Disruption to the material flow of pork will also lead to socio‐economic implications for communities in regional Australia in which pork is produced, for example, financial and mental distress related to loss of job security and unemployment.

## Discussion and conclusion

This study contributes to the literature and practice through its provision of detailed information about the pork supply chain structure, processes and vulnerabilities, which is fundamental to comprehend the implications of restrictions on the material flow in supply chains. The findings of this study offer a basis for future quantitative modelling and economic assessments of EAD control strategies.

The study highlights that Australia's pork supply chain is dominated by a small number of vertically integrated businesses operating under a low redundancy, full asset utilisation model. Similar structural features and concentration within the pork supply chain have been observed in other countries.^21^, ^22^ While efficient under normal conditions, this structure limits flexibility during disruptions such as EAD outbreaks.^23^, ^24^

Findings of this study highlight that regulatory controls on material movement – such as restrictions on semen, live animal transport or abattoir operations – can trigger cascading socio‐economic impacts. These include loss of income, job insecurity, animal welfare concerns and reputational damage. Importantly, not all impacts are financial; psychological stress and public trust erosion are likely to be significant, though considerably more difficult to quantify. This suggests that cost–benefit analyses – which are often used to quantify the impact of EADs – alone are insufficient for guiding disease response decisions.

Furthermore, disruptions in the pork supply chain may also affect other meat sectors. For example, due to less pork being available in the domestic market, subsequent higher pork prices and a perception that the pork is diseased or less wholesome, consumers may shift their demand towards other animal protein sources, for example, chicken, beef or lamb.^25^, ^26^ The increased demand for pork substitute meat products could cause pressure on these meat supply chains, which potentially may lead to an increase in prices of these products should the meat supply not be sufficient. Conversely, EAD outbreaks in other meat sectors or diseases affecting multiple species could also impact pork or wider protein demand and logistics.

To mitigate these risks, several recommendations emerge. First, context‐aware decision‐making is needed. This means that government authorities, for example, Chief Veterinary Officers, must weigh disease control benefits against the socio‐economic costs of movement restrictions.

Furthermore, the findings demonstrate a relatively large range of stakeholders directly involved in the pork supply chain will likely be affected by movement control measures as a response to an EAD event. Implementing effective disease control measures that minimise the social‐economic implications requires close collaboration between government authorities and stakeholder groups within the pork supply chain. There is a need to find a balance between disease control imperatives and minimising supply chain disruptions. Zero disruption is not possible, but consideration of broader risks and impacts may minimise unnecessary disruption without compromising disease control integrity.

The development of effective collaborations needs to be a central part of the preparedness planning process for disease events. Such collaborations will not only broaden all stakeholders' awareness of pork supply chain processes and improve decision‐making, but it will also contribute to minimising the loss of stakeholders' confidence in one another, fostering a sense of shared ownership, integrating different perspectives and experiences and helping to support public trust in the management of animal disease events. In practice, this means that representatives of key stakeholder groups (e.g., semen provider, key commercial pig producers, pork industry organisations, logistics providers, abattoirs, renderers, retailers and government authorities) should be involved in the process of coproducing EAD preparedness plans that consider a broad range of risks and impacts (e.g., working groups that develop strategies).

Collaborative EAD preparedness planning also needs to identify policy, strategy and investment opportunities to limit the identified potential implications from disruptions in the material flow and to minimise the scale and duration of disruptions. This may include for example, nuanced consideration of the balance of risk mitigated and created by different movement control approaches, mental health support programmes for stakeholders who are affected by stress due to destruction of animals (e.g., producers/farm workers, veterinarians) and temporary aid programmes that assist key stakeholder in minimising their financial loss. Such temporary support programmes were rolled out in other countries to minimise socio‐economic implications due to EAD responses.^25^, ^27^

The implementation of declared areas and movement controls creates tension between disease control and supply chain continuity. Ad hoc large spatial scale implementation of movement controls (e.g., material flow standstill in one or several states or territory) may not always be necessary, but uncertainties about EAD spread risk can trigger these, causing an unnecessary scale of socio‐economic implications.

Disease control measures are driven by disease epidemiological considerations. To overcome the uncertainties about EAD spread risks, government and pork supply chain stakeholders need to invest in secure and interoperable digital platforms to support epidemiological surveillance.^28^ This will assist decision‐makers to better trade off the EAD spread risk against the socio‐economic implications of movement control measures.

Clear and effective communication plans that target supply chain stakeholders and the public build trust and confidence in stakeholders' EAD preparedness and response strategies. Most importantly, they address consumers' concerns (e.g., perception of human health risks).^11^ These plans need to be maintained to support public and consumer trust and confidence in government authorities and the pork industry. This will be important for a swift recovery from an EAD event.

There is value in exploring biosecurity risks across all supply chain segments and developing models that integrate existing traceability data (e.g., PigPass) with disease spread simulations (e.g., Australian Animal Disease Spread (AADIS) Model^29^). Enhanced data sharing from downstream stakeholders would improve real‐time, sophisticated modelling of material flows in disease scenarios and support evidence‐based policymaking. Further quantifying the socio‐economic impacts of different control strategies would further support evidence‐based policymaking.

A limitation of this study is the geographic concentration of participants in Queensland and New South Wales, and the absence of input from major retailers. These gaps may affect the generalisability of downstream insights.

In conclusion, understanding the structure and vulnerabilities of the pork supply chain is essential for designing disease control measures that are both effective and socially responsible. This study will help disease control regulators to better understand the pork supply chain consequences that might arise following risk management decisions and lead to balanced and collaborative EAD preparedness and response strategies.

## Conflicts of interest and sources of funding

This study was commissioned and funded by the Queensland Government, Department of Primary Industries and the New South Wales Government, Department of Primary Industries and Regional Development. Kirsty Richards and Robert van Barneveld are representatives of the pork industry in Australia who may potentially be affected by regulatory control measures in the case of an emergency animal disease outbreak. This association could potentially have affected the objectivity of their contributions. The other authors declare no conflicts of interest for the work presented here.

## Supporting information


**Data S1.** Supporting information.

## Data Availability

The data collected for this study are unavailable since participants have not consented to sharing the raw data with third parties.
