# Supplementary material for: Description of the Australian pork supply chain and implications for national biosecurity management
Source: Aust Vet J. 2025 Sep 13;103(9):524–32. doi: 10.1111/avj.70011 (PMC12444612; doi:10.1111/avj.70011)
Supplement: Supplementary file 1 — Data S1. Supporting information. [file AVJ-103-524-s001.docx]

**Supplementary material**

*“Description of the Australian pork supply chain and implications for national biosecurity management”*

This document provides supplementary material which should be considered in conjunction with the main article.

**1 Detailed description of the Australian pork supply chain supply**

**1.1 Upstream material flow**

The material flow within the pork supply chain (see Figure 1 in the main article) starts with semen supply from boar studs. In Australia, over 90% of commercial pig breeding relies on artificial insemination using domestically produced, fresh semen from boar studs (Animal Health Australia, 2023). The pig industry depends solely on regular deliveries of fresh semen, viable for only a few days, as commercially viable freezing methodologies for on-farm storage and embryo transfer technologies remain infeasible. Only a small proportion of pig farms, mostly small commercial, smallholder and pig-keeper farms, use natural mating as a reproduction method.

Fresh pig semen is delivered from boar studs to commercial farms and multiplier herds two to three times a week. Some nucleus herds may also operate an internal boar stud. These nucleus herds are breeding herds used to produce genetically superior pigs and are typically closed breeding systems, i.e., no external pig intake.

Multiplier herds source their gilts (female pigs that have not yet farrowed) from a nucleus herd and semen from a boar stud, to produce gilts for commercial pig farms. Sows in multiplier herds produce up to 6-7 litters during their productive life, after which they, along with unselected progeny pigs, are sent to abattoirs for slaughter.

Groups of sows are artificially inseminated each week, resulting in pigs being born in weekly cycles. This process is known as continuous farrowing. A minority of farms practise batch farrowing, where groups of sows are inseminated and farrowed in batches every 4-6 weeks. After gestation and farrowing, piglets are weaned at the age of 3 weeks. ‘Weaners’ (non-suckling pigs weighing between 6 to 30 kg, 3-10 weeks of age) progress to become ‘growers’ (pigs weighing approximately between 30 to 70 kg) at approximately 10 weeks of age, then transition to the ‘finisher’ (weighing between 70 and 100+ kg) stage at approximately 17 weeks of age, before sales around four to eight weeks later (Australian Pork Limited, 2017).

Progeny pigs may be raised in an ‘all-in-all-out system’, where same-aged pigs are grouped from weaning until sale. This is considered a superior practice for the management of pig health. More commonly, progeny pigs are reared in a continuous flow system involving a continuous entry and exit of pigs through a flow/site. At the end of their productive life, sows are replaced by gilts from a multiplier herd, although some commercial herds can self-replace sows through internal multiplication. Approximately 60% of breeding sows in a commercial pig herd are replaced annually.

All production activities, i.e., breeding to finishing, may occur on one site. Such sites are known as farrow-to-finish farms. Some farms only undertake breeding, gestation, farrowing and lactation functions, producing weaners that move to other commercial farms at weaning (East, Davis, et al., 2014). Weaners may then remain on one site through to finishing or may move from a weaner site to a grower and/or finishing site. Large commercial pig operations may handle a range of different pig flows including farrow to finish, weaner and owned or contractor grower/finisher farms. These large operations may also integrate the management of boar studs, nucleus and multiplier herds, abattoirs and feed mills. Pig growing enables contractors to diversify, build equity and secure long-term income. These contract pig farms are typically required to comply with the production standards, e.g., specific finishing time and weight, set by their customers.

Commercially grown pigs are transported (via internal and external live animal logistics providers) up to three times in their lifetime, depending on the type of production system. Pigs are moved at weaning stage from farrowing to weaner location, then to grower-finisher location, and finally the pigs are transported to the abattoirs. Commercial farms also infrequently provide pigs to universities and hospitals for research and development purposes.

The process from breeding to finishing in the commercial pig farm system takes about 40 weeks, i.e., 1 week for breeding, 16 weeks for gestation, and 22 weeks for progeny to progress through farrowing/lactation, weaning, growing, and finishing phases to sale.

Small commercial/smallholder/pig-keeper farms mostly use natural mating for reproduction. Activities at these farms include breeding, grow-out and finishing of pigs. For breeding at small commercial/smallholder/pig-keeper farms, boars may be moved from and to different farms for mating (periodic arrangements). Grow-out time for pigs to sale in these farms can vary from the typically meticulous timing of larger commercial pig farming system.

Pig producers in small commercial/smallholder/pig-keeper farming systems typically buy breeding sows and boars, weaners and growers from other farms, including small commercial farms or the saleyard (East, Davis, et al., 2014; Schembri et al., 2015; Schembri et al., 2010), if saleyards are available, as in the case of New South Wales. Anecdotal evidence suggests that about 5% of pigs were sold in New South Wales through saleyards a decade ago. However, with the closure of several saleyards in recent years and only one pig saleyard remaining in New South Wales, the number of pigs sold through saleyards has likely decreased as well (no official records available).

Smallholders and pig keepers are increasingly using online platforms like Gumtree^^[[1]](#footnote-1)^^ and Farm Tender^^[[2]](#footnote-2)^^ to sell pigs, with most sales on these platforms being unregulated and lacking prior veterinary assessment. As a result, buyers often receive limited information on the animal's health, and there are no official records of the number of pigs traded through these platforms.

Live animal logistics providers are trucking companies that transport live pigs from one point in the upstream supply chain to the next, though some pig producers transport their own pigs. In all cases, both the pig producer and transport driver are required to visually inspect and ensure animals are fit for the journey and to record and report movements. Logistics operators regularly transport either full loads (averaging 270 to 342 pigs) from one property or split loads across two to three different properties to abattoirs. These operators also regularly transport groups of pigs from property-to-property for growing out. Pigs are transported separately from other livestock species in specialised trailers designed for their needs, including welfare considerations such as cooling in hot weather. Integrated pork businesses that operate boar studs, nucleus and multiplier herds, commercials farms and abattoirs, may use both internal and external logistics providers for moving live animals (Hernández-Jover et al., 2008). After delivery, trucks used to move pigs from commercial farms are usually cleaned before the next consignment.

Less frequently, short-term property-to-property transfers may also occur at the farm level. These transfers often involve small-scale farms borrowing boars for breeding purposes (Schembri et al., 2015). Property-to-property transfer between small-scale farms may also occur where animals are traded between farms. These movements also occur less frequently than regular commercial movements between associated properties within a pig flow and to abattoirs.

Pigs are a popular feature at some agricultural shows in Australia. For these shows, pigs are typically moved from small commercial/smallholder/pig-keeper farms to the location where the agricultural shows are taking place (mostly intrastate movements). Transportation is handled by logistics providers or directly by pig producers.

All property-to-property and property-to-abattoir movements of live pigs are required to be recorded by dispatchers and reported to the PigPass^^[[3]](#footnote-3)^^ database which is the national pig traceability system. Information that is recorded in the PigPass database includes the national vendor declaration serial number, property identification code (PIC) and address, and body tattoo identifier of an animal.

**1.2 Midstream material flow**

Within the midstream stage of the pork supply chain, the flow of materials commences at an abattoir, also known as a meat processor, slaughterhouse, or meatworks. This is a facility where animals are slaughtered for food/meat production and serves as the main actor in the midstream segment of meat supply chains. In Australia, abattoirs must be accredited by the state/territory food safety authority to slaughter and process meat for the domestic market, and with the Australian Government where product is exported. Export-certified abattoirs adhere to additional quality specifications beyond the standard food safety controls, e.g., importing country requirements.

However, export-certified abattoirs also supply substantial quantities of meat to the domestic market and some domestic customers of abattoirs (e.g., specific retailers) require pigs to be processed at export-certified facilities. Australia currently has seven export-certified pork abattoirs that process about 90% of pigs nationally. Most of these abattoirs are part of an integrated supply chain model and include breeding, growing and processing of pigs within their business operations. In addition, there are five domestic abattoirs that collectively process about 10% of pigs produced in Australia.

At the export-certified abattoirs, pigs arrive by trucks, are off-loaded in groups into lairage pens. In lairage, pigs undergo an ante mortem health inspection by an Australian government veterinarian, with most receiving clearance for unconditional slaughter and proceeding to normal processing. Some pigs are given the disposition of conditional slaughter, which may include identified pigs that are held and sent for processing last, or emergency kills. Some pigs may also be condemned and euthanised in lairage, with their bodies sent to a renderer (details below).

Healthy pigs spend an average of two hours and a maximum of 24 hours in the lairage at export-certified abattoirs before they are slaughtered. Progeny pigs are usually stunned in small groups in a stunning chamber before slaughtering (Jongman et al., 2021). Abattoirs produce a range of materials which include primary meat products, e.g., carcase, trimmed meat for human consumption, offal (edible and inedible), non-offal materials (by-products such as blood, bone, hair) and other materials (e.g., biological specimens, see below).

After slaughtering, the carcases are scalded, de-haired, singed, and polished. The pig carcase market for Australia is skin-on. Hence, the skins are not removed from the carcases. Prior to evisceration, by-products like hooves, horns, and blood are removed and sent to a renderer. Each carcase is given a unique identification number that is recorded in the abattoirs’ database and able to be matched with a PigPass record including national vendor declaration serial number, property identification code (PIC) and body tattoo identifier. The evisceration process involves separation of red and white offal from the polished carcases. Carcase, red and white offal are inspected by Australian government-authorised officers at postmortem inspection, graded, and weighed. The processes from stunning to chilling of the carcase takes about two hours.

Carcases are moved to a chilling room (snap or blast chill at around 4^o^C) and kept there for 12 to 24 hours to enable muscle relaxation and a decrease in the pH level of the meat. Achieving a pH level of 5.6-5.8 is crucial for enzymatic processes that tenderise the pork meat on the bone before the meat is removed from the carcase. The whole or split carcase can be kept in a chiller (cold rooms) at the abattoir for up to five days. From the chiller, carcases can be moved to either an export-accredited boning room which is integrated into the abattoir facility, or offsite to an external export or non-export certified boning room. Within boning rooms, the meat is separated from the bones of the carcase. In a service kill arrangement between abattoirs and their customers, known as operators, abattoirs slaughter and process the animals to an agreed specification. Fresh pork cuts and/or whole carcases are then dispatched or delivered to the operators’^^[[4]](#footnote-4)^^ preferred destinations. The abattoir may have oversight over dispatch information, or the operator may collect from the abattoir at which point the abattoir loses visibility over onward movement. Operators may be integrated with farms and abattoir(s) or operate independently. Large supermarket chains like Woolworths, Coles, and Costco source their pork exclusively from export-accredited abattoirs through designated operator(s). Other retailers, e.g., ALDI and IGA, source their pork products from either export-accredited or domestic-orientated abattoirs through operators.

Whole or split carcases may be supplied from an abattoir’s chillers to boning rooms, wholesalers’ chillers or cold stores. Products in cold stores may be exported (if processed at an export-accredited abattoir), distributed to the domestic retail market, or moved for further processing at a value-adding processor.

Pork cuts leaving boning rooms are distributed by refrigerated trucks to downstream supply chain entities including cold stores (for export or domestic distribution), wholesalers’ chillers, value-added (further) processors, retail value addition facilities (Coles/Woolworth/Costco only) or a retail packer (Coles/Woolworth/Costco only).

The only product derived from pig processing that is owned by an abattoir is the offal (see Figure 1 below). Red offal and white offal (including mucosa, casings, rectum, and uterus) are cleaned, packed, and transferred to cold stores where they are accumulated for export. Some red offal is distributed to the domestic market through wholesalers and butchers. Pork cuts and offal designated for export may be redirected to domestic retail but require re-stamping in accordance with Australian government regulatory requirements to do so. Some co-products such as low-quality trimmings, ears, and flare fat are transferred to pet food producers. Abattoirs may send some by-products (e.g., blood, pancreas) to biochemical users, like research and pharmaceutical institutions, however most co-products and by-products are commercially used either by abattoirs or by customers such as renderers or pet food processors.

Some by-products that are produced during processing such as blood require specialised transportation tanks, while other by-products and co-products are transported to renderers via large trailers or to pet food producers via refrigerated trucks. Co-products like hearts, tongues, and kidneys are packed similarly to primary meat products and distributed to downstream entities. Refrigerated logistics providers transport processed meat products including carcases, boxed pork cuts, and boxed co-products (head, kidneys, livers) from abattoirs and downstream actors to other supply chain entities in a refrigerated environment, either frozen or chilled. The distances between abattoirs and downstream domestic supply chain entities vary but can exceed 12 hours. High volumes of edible product are distributed at regular frequencies across and between states.

Meat products that are produced in abattoirs include primary meat products (edible), co-products, and by-products. Co-products are materials derived from carcases that are intentionally produced along with primary meat products. These materials are secondary products that have a significant economic value. Some are suitable for human consumption, such as hearts, livers, and tongues. By-products are materials derived from carcases that are not suitable for human consumption or are not typically produced for human consumption, for example blood. These products were partially included in the mapping of material to illustrate the link between human and non-human food supply chains, including stakeholders who may be affected by disease control measures.

Figure 1 offers information about the different types of materials produced by pig abattoirs.


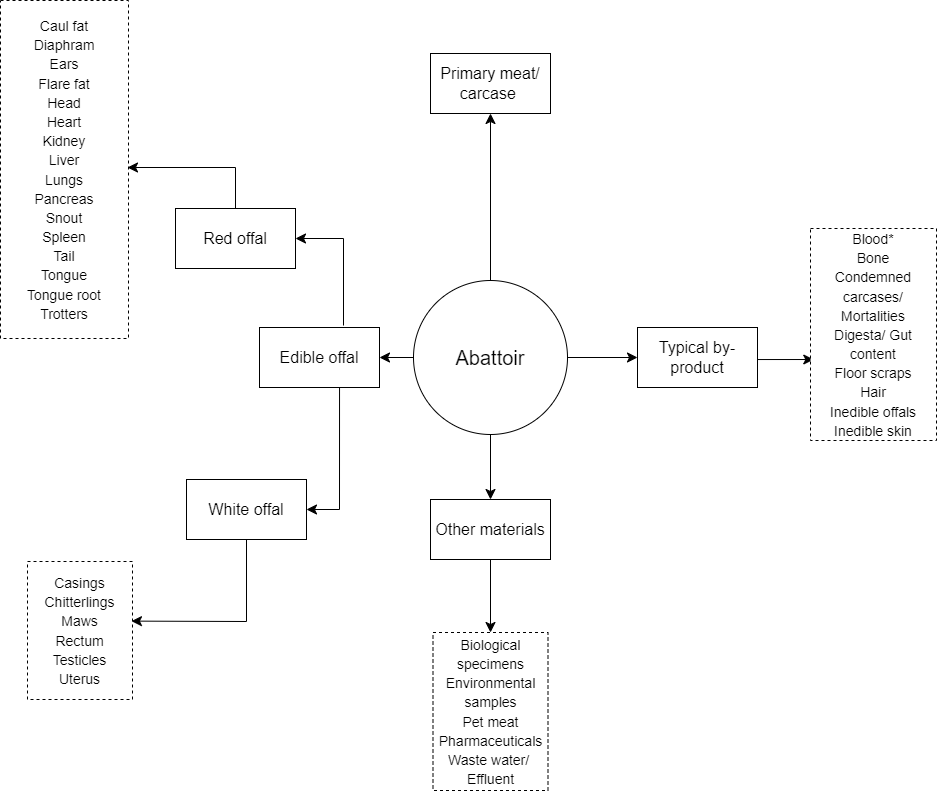


Figure 1: Categorisation of materials produced by pig abattoirs. Note: Edible offal is considered as a co-product. * While animal blood is generally suitable for human consumption, it is typically a material that is treated by abattoirs as by-product.

In a service kill arrangement between abattoirs and their customers, known as operators, abattoirs slaughter and process the animals to an agreed specification. Fresh pork cuts and/or whole carcases are then dispatched or delivered to the operators’^^[[5]](#footnote-5)^^ preferred destinations. The abattoir may have oversight over dispatch information, or the operator may collect from the abattoir at which point the abattoir loses visibility over onward movement. Operators may be integrated with farms and abattoir(s) or operate independently. Large supermarket chains like Woolworths, Coles, and Costco source their pork exclusively from export-accredited abattoirs through designated operator(s). Other retailers, e.g., ALDI and IGA, source their pork products from either export-accredited or domestic-orientated abattoirs through operators.

Pork cuts and offal designated for export may be redirected to domestic retail but require re-stamping in accordance with Australian government regulatory requirements to do so. Some co-products such as low-quality trimmings, ears, and flare fat are transferred to pet food producers. Abattoirs may send some by-products (e.g., blood, pancreas) to biochemical users, like research and pharmaceutical institutions, however most co-products and by-products are commercially used either by abattoirs or by customers such as renderers or pet food processors.

Rendering is the process of managing by-products from the carcase processing, using heat to transform inedible offal as well as condemned carcases of injured/sick animals into secure, pathogen-free feed proteins and other valuable by-products. At the renderer, by-products are cut, grounded into small pieces, blended, and cooked to produce about 50% wastewater, 30% protein meals (bone, blood) and 20% fat (oil, lard, and tallow). The high-protein meals from rendering are used to produce domestically distributed dry pet food, e.g., kibble, meat and bone meal, tallow (rendered fats), and oils and oleochemicals, e.g., cosmetics, candles, soaps. Protein meal is also exported.

Pet food processors use by-products and co-products from animal processing at abattoirs and from renderers to produce wet or dry pet food for both exported and domestic markets.

Knackeries are a distinct type of meat processor in Australia that slaughter animals exclusively to produce animal feed, but not for human food consumption and were therefore not further considered in this study.

**1.3 Downstream material flow**

Downstream activities within the supply chain include the distribution of meat from the abattoir (processor) level through value-added processors and retailers to the final consumer. Value-add processors are secondary meat processing facilities that receive pork cuts from boning rooms and further process them into products like bone-in hams, bacon, sausages, and marinated pork. These products are then packaged and supplied to supermarket chains such as Woolworths and Coles, or other retailers like Costco, ALDI and Metcash. The pork products, which for some retailers (Coles, Woolworths, Costco) must originate from an export-certified abattoir or boning room, can remain at the value-added processors for up to five days.

Other secondary meat processing facilities owned by large supermarket chains (Woolworths and Coles) include retail packers and retail value adders. These receive pork cuts exclusively from export-certified abattoirs or boning rooms. Retail packers process these cuts according to the retailer’s specifications and package them for distribution. Retail value adders further enhance the products, for example by adding herbs, infusing moisture, or marinating, before packaging and sending them to supermarket locations.

Wholesalers source and sell meat products from operators in bulk, often storing them in large cold stores or chillers. They may also act as operators. Wholesalers receive whole or split carcases from abattoirs and distribute them to butchers or send them to boning rooms for further processing. Pork cuts and red offal are also stored by wholesalers for up to five days before being transported to butchers, retailers like ALDI and Metcash, or food service providers.

Butchers may receive whole or split carcases through operators or wholesalers from abattoir or wholesaler chillers and process them into pork cuts according to customer specifications. Butchers can also obtain pork cuts and red offal from wholesalers, add value through further cutting and packaging, and supply other retailers. Additionally, butchers may operate directly at the retail interface with consumers.

Pork cuts are also moved from boning rooms to cold stores where the products can be stored to supply the domestic meat market, e.g., for increased demand around Christmas, before they are transferred to a value-added processor or retailer markets. According to the [standards and guidelines of shelf-life for meat products](https://www.primesafe.vic.gov.au/standards-and-guidelines/primenotes/shelf-life-and-labelling-requirements-for-meat-products/), frozen pork cuts can be stored for about ten months (PrimeSafe, 2024).

The export of pork products involves the use of cold storage facilities for consolidating and in some cases re-packing goods destined for export. Cold stores used for this purpose are typically located near airports or seaports and may also store products for domestic markets. Pork cuts and offal designated for export may be redirected to domestic retail but require re-stamping in accordance with regulatory requirements to do so. Pork export volume is relatively small with approximately 12% of pork products being exported in 2022 (ABS, 2023) (see below). However, exported product is an important and valuable component of the supply chain.

Pork meat retailers are categorised into three groups: supermarket chains like Woolworths and Coles, other retailers such as Costco, ALDI, Metcash, IGA, and food services like restaurants and meal-kit deliveries. Woolworths and Coles operate a ‘whole pig model’, sourcing pigs from specific farms that comply with their standards. Operators have these pigs processed at export-certified abattoirs and meat is then further handled by supply chain entities, including value-added processors and retail packers, under the supermarket chain's ownership. The other type of retailers source pork as cuts from operators who process pigs at export-certified or domestic abattoirs, via cold stores, butchers, wholesalers, or value-added processors. This approach is less centralized and more flexible than that of Coles and Woolworths but involves less oversight of animal production and meat processing. Waste from these retailers is either sent to landfills or to renderers.

Domestic consumers purchase meat products or co-products from retailers, e.g., supermarkets or other retailers based on their food preferences. For home consumption, consumers or delivery services transport the meat products from the retail store to the consumer’s home, where the meat products undergo their final processing and consumption or are discarded as waste (link not shown in Figure 1, main article).

**2 Additional descriptive statistics about the Australian pork supply chain**

Table 1 provides information about pig production volume by state/territory in Australia. Queensland and New South Wales together produce approximately 50% of the pigs produced in Australia.

Table 1: Average state-level pig production (2021)

| State/Territory | Annual pig production volume  (‘000 heads/numbers) | Proportion of national pig production (%) |
| --- | --- | --- |
| Queensland | 754.9 | 29.3 |
| New South Wales | 543.3 | 21.1 |
| Victoria | 496.9 | 19.3 |
| Western Australia | 391.4 | 15.2 |
| South Australia | 379.7 | 14.7 |
| Tasmania | 11.3 | 0.44 |
| Northern Territory | 0.04 | <0.01 |

Source: ABARES (2022). Note: Data beyond 2021 consists of forecasted information only and was therefore not included.

Table 2 offers an overview of the average number of slaughtered pigs at the state/territory level across Australia in 2023^^[[6]](#footnote-6)^^. Queensland recorded the highest average number of slaughtered pigs nationally, followed by Victoria and South Australia. Together, the three states processed 69% of slaughtered pigs nationally.

Table 2: Quarterly average state-level slaughtered pigs (2023)

| State/Territory | Quarterly pig slaughtered (‘000 heads/numbers) [Mean ± SD] | Quarterly mean % of national pig slaughter |
| --- | --- | --- |
| Queensland | 362.6 ± 8.8 | 25.0 |
| Victoria | 326.3 ± 10.9 | 22.5 |
| South Australia | 305.7 ± 15.7 | 21.1 |
| Western Australia | 232.8 ± 9.3 | 16.0 |
| New South Wales | 217.6 ± 6.9 | 15.0 |
| Tasmania | 6.8 ± 0.4 | 0.5 |
| Northern Territory | - | - |

Source: Australian Bureau of Statistics (2023). Note: SD for standard deviation.

**3 Pork export statistics**

In 2021, Australia exported 11.2% of the total pig meat production (Australian Bureau of Statistics, 2023). Figure 2 shows that the minimum and maximum export proportions of pig meat from Australia between 1990 to 2021 ranged from 2.3% to 22.5% (Australian Bureau of Statistics, 2023). These proportions correspond to annual quantities ranging from 7.65 to 91.51 kilotons, with an average annual export volume of 43.6 kilotons (Australian Bureau of Statistics, 2023), as depicted in Figure 2.


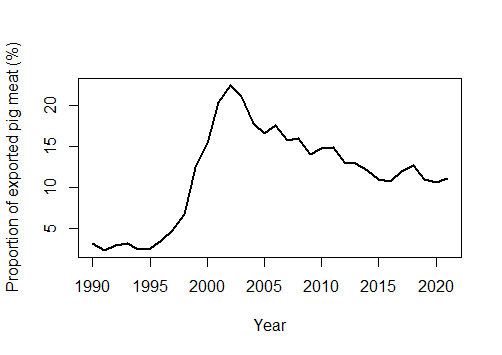


Figure 2: Proportion of pig meat exported from Australia. Source: Australian Bureau of Statistics (2023).


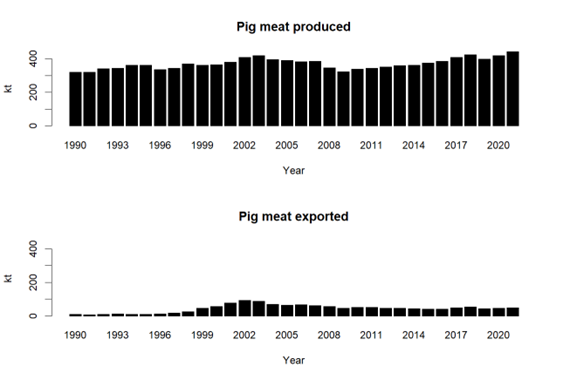


Figure 3: Australia’s pig meat production and export. Source: Australian Bureau of Statistics (2023).

**4 Loss calculation due to disrupted semen supply**

The following assumption for the loss calculation due to disrupted semen supply were taken:

- the loss is irretrievable (lost matings cannot be recouped and it is not possible to re-insert an entire week’s worth of unmated sows back into the herd at a subsequent cycle prior to the next intended mating some 20 weeks away),
- 10 pigs per lost mating not produced, with each mating including two doses of semen,
- sows will be non-productive and need to be prematurely culled when able to be permitted to move and abattoir has capacity to process.  It is expected in an EAD scenario where there are disruptions to semen supply that there are likely to be delays in live pig movements too,
- to fill the gap at the next mating cycle created by culling non-productive sows (due to missed mating), additional replacement gilts will need to come in sooner than planned, and
- genetic lag has been excluded from loss calculations.

The financial loss calculation due to disrupted semen supply was estimated as follows:

***LOSS PER SOW***

1. Per lost mating lost income and additional cost to producer

- 10 lost pigs * 80kg dressed weight/pig * $4.10/kg (the price may fluctuate) = $3,280 lost income to producer and 800kg meat lost to the supply chain,
- $32.00/week feed for non-productive sows until able to be culled – assume one month lag until able to be culled = $140 feed cost,
- $650 replacement gilt cost,
- Total: $3,280 +$140 +$650 = $4,070

1. Per lost mating lost income to transporter with no pigs to transport

- $9 average per pig to transport to abattoir * 10 pigs = $90

1. Per sow lost income to boar stud

- 2 doses of semen/sow * $12 each = $24

1. Per lost mating lost offal income to abattoir

- ~$10/pig offal * 10 pigs = $100

1. Total lost income and additional costs per missed sow mating from #1-4 above: $4,070 (producer)+$90 (transport) +$24 (boar stud) +$ 100 (abattoir) = $4,284, with meat lost from the supply chain = 800kg (10 lost pigs per sow).

***LOSS PER 500-SOW HERD PER WEEK***

The estimate loss for 500-head sow herd is based on the estimated loss per sow (see above):

- Assuming 25 sows from the herd farrow each week
- 25 sows * $4,070 = ~$107,000/week collective income loss across the up and mid streams of the supply chain.
- 250 lost pigs * 800kg per pig = 20 tonnes meat lost from supply chain

**5 Information flow within the pork supply chain**

This section focuses on the information flow or traceability of materials handled within the Australian pork supply chain. Material traceability within the supply chain is the ability to follow the movement of the material, e.g., semen, animals, or meat products through all steps in the supply chain, both forward and backward. Data transmission relates to the information that is shared among the stakeholders to facilitate the material flow and traceability within the supply chain. Figure 4 illustrates the information flow within the pork supply chain.

Similar to the material flow in the pork supply chain (see Figure 1 main article), the information flow within the chain commences at the boar stud where information about each boar, date and time of semen collected is recorded. This information is also provided on the label of each semen batch that leaves the boar stud towards multiplier and commercial breeding herds. Only the boar stud and the receiver of the semen, e.g., multiplier herd and commercial pig farm have access to this information. Pig producers share pig performance data after insemination with breeding companies via the [PigBLUP](http://agbu.une.edu.au/pig_genetics/pigblup.html)^[[7]](#footnote-7)^, which is a genetic evaluation system (PigBLUP, 2022).

Australia’s live pork supply chain relies on batch (or mob-based) traceability (Meisinger et al., 2008), wherein the data on the flow (movement) of pigs before slaughter can be traced back to groups of pigs at the originating property, not usually to individual pigs other than cull breeding stock.

Across Australia, land on which pigs are kept must have a PIC. All pig movements between different properties (PICs) must be recorded in the national PigPass^[[8]](#footnote-8)^ database, which is immediately transferred to Australia’s National Livestock Identification Scheme (NLIS) database.

PigPass is Australia’s national vendor declaration and tracking system for live pig movements. The PigPass system provides near real-time information about the movements of all live pigs in Australia (Hernandez-Jover, Schembri, et al., 2008). The PigPass system is hosted by Australian Pork Limited (APL), the national peak organisation of the pork industry.

Every pig <25kg (Queensland <30kg) moving off a property in Australia is required to be identified with an NLIS ear tag. Pigs >25 kg (Queensland >30 kg) that move off a property must be identified with a tattoo brand or NLIS ear tag, excluding pigs in South Australia moving between PICs where ownership does not change. The tattoo brand is a unique sequence that varies in composition by state, e.g., 6-number sequence (for New South Wales) 3-letter and 1 number (for Queensland), 3-letter (for South Australia). Each brand is associated to a specific PIC, thereby facilitating traceability. When a movement is undertaken, both PIC and tattoo brand are recorded on PigPass documentation and in the PigPass database. At the abattoir, tattoo brands are the means by which individual pigs are identifiable to the consignment they originated from. Consequently, the legibility of branding (Hernandez-Jover, Schembri, et al., 2008) and the use of registered brands (Hernandez-Jover, Wu, et al., 2008) are crucial for traceability.

The receiver of pigs moved between different properties (PICs), e.g., abattoir, other pig producer/property, pet pig owner, saleyard, show event, etc. must acknowledge the movement in the PigPass system by using the senders/originating serial number from the PigPass National Vendor Declaration (NVD). At saleyards, the sale transaction information on the pigs sold (number and description) are recorded for each pen. Unrecorded or unidentified cash transactions which often occur at the saleyard lead to information loss which poses a threat to the traceability of live pigs (Schembri et al., 2006; Sithole et al., 2009).


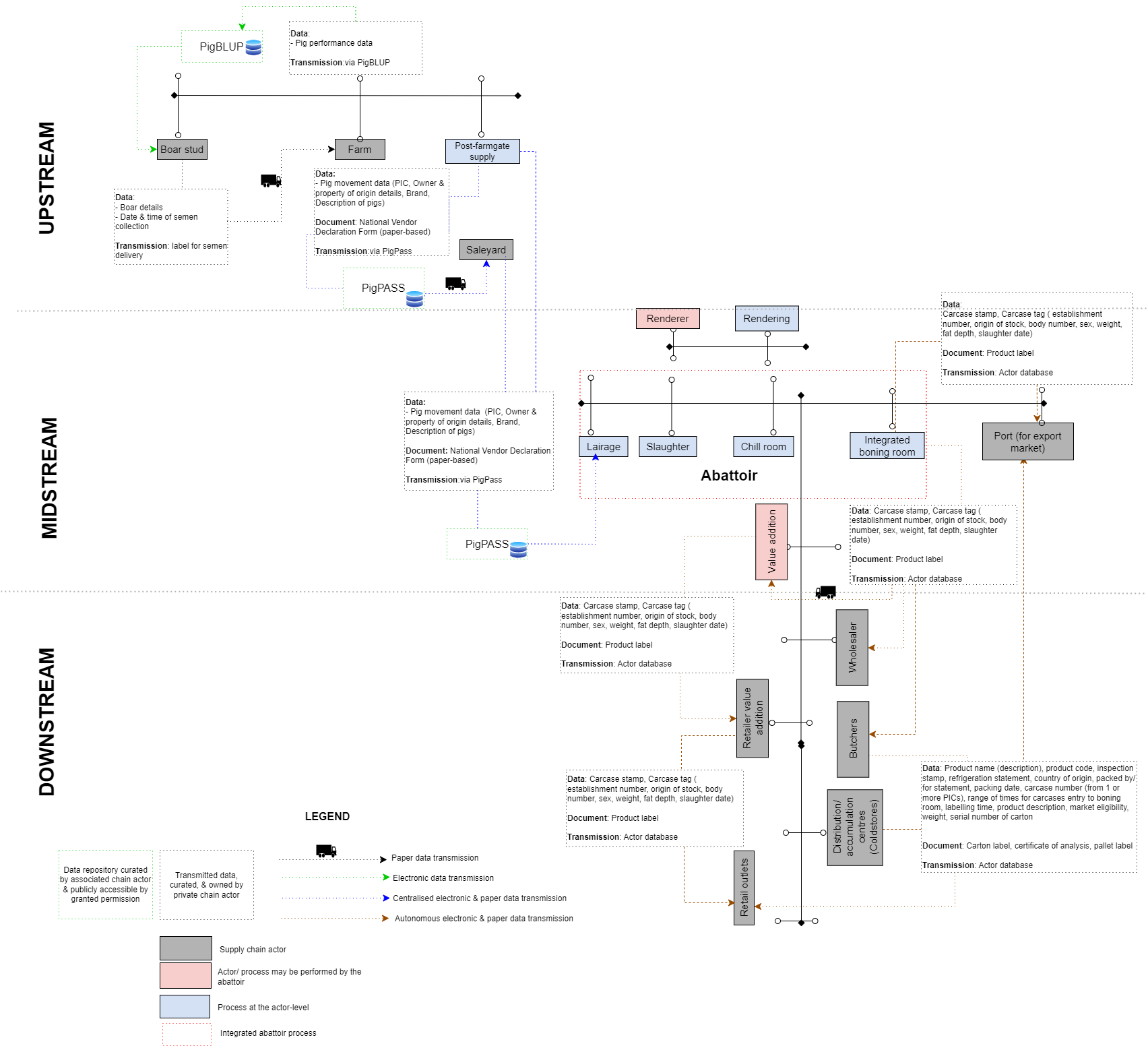


Figure 3: Data transmission within Australia’s pork supply chain

The PigPass NVD is a paper-based (usually) declaration form that facilitates the tracking of animals' movement. A person responsible for the pigs at the property of origin completes parts A, B and C of the PigPass NVD prior to the pig transport driver completing part D and the destination Part E. For transactions at the saleyard, the PigPass NVD is commenced by the vendor and a copy given to the transporter, who completes Part D and provides a copy to the purchaser/receiver. The movement details are then registered in the PigPass database by operators of the saleyards. For property-to-property movements other than where exempt, e.g., movements between the same PIC, movements in South Australia between PICs under the same ownership, the recipient registers the PigPass details in the PigPass database.

The [PigPass NVD](https://australianpork.com.au/sites/default/files/2021-06/PigPass-NVD-attachment-page-only.pdf) captures the following: (i) serial number of the PigPass NVD, (ii) the PIC, (iii) owner and property of origin details, (iv) brand/tattoo information, (v) description of pigs (including the number of pigs, sex, type of pig) destination, special risks and identifications, time pigs have resided on the property, chemical treatment information, piggery and feed quality assurance information, transporter and receiver information.

PigPass data are not publicly available. Restricted information about pig movements can be accessed by supply chain stakeholders with a PigPass account. Subject to strict conditions and appropriate justification for access, APL may offer access to information that is held within the PigPass database upon request from stakeholders who are not directly involved in the material flow.

Transportation routes are not registered in the PigPass. Although most transportation providers use a geographical position system (GPS) for route tracking/recording and vehicle monitoring, this information is not usually shared with other stakeholders in the supply chain and is only accessible to the individual logistics provider. Furthermore, logistic providers generally only record the required details for Part D in the PigPass and hence animal records are linked to a brand. Thus, supply chain visibility is generally linked to batches of supply between two properties, and not individual animals. Some transporters use their own paperwork alongside PigPass documentation. Additionally, either the transporters or farms may identify individual animals. Multiple brands (tattoos) can be involved in a movement; hence brands can sometimes be used to distinguish groups within the larger movement. In the event of an immediate mandated livestock movement standstill, real-time data about animals in transit may require contact with pig properties, transport providers and abattoirs, to provide data on the location of animals in transit at a specific point in time.

For property-to-abattoir movements of pigs, the operator that is buying the pigs books the pigs in with the abattoir and the abattoir prepares a kill agenda including all groups (lots) that are booked in. The operator coordinates with the abattoir on the scheduled delivery time for each lot, based on the kill agenda (email, phone call, text message). Either the operator or the producer then coordinates with a livestock transport provider to transport the pigs to the abattoir (email, phone call, text message). Transporters may also coordinate with the abattoir to align transport scheduling needs with processing schedules. Both abattoirs and transport operators prepare schedules for activities to be undertaken the following week. A person responsible for the pigs at the property of origin completes parts A, B and C of the PigPass NVD prior to the pig transport driver completing part D and the abattoir Part E. The abattoir then registers movement in the PigPass database after pig processing.

Each PigPass consignment is known as a lot. As there may be pigs for different markets within the same consignment, lots may include sub-lots. Pigs within a lot, or if there are sub-lots, within each sub-lot, will all share the same body brand/tattoo. At unloading, pigs are boxed into pens according to the lot kill agenda. An *ante mortem* card is allocated to the front and back of each pen. These cards include *ante mortem* inspection information that assists correct reconciliation of numbers of pigs within lots/sub-lots. For example, if 40 pigs were booked, consigned, and included on the PigPass but two were deemed suspect at *ante mortem* inspection and held for later processing, the *ante mortem* card would clarify why only 38 pigs from the booked lot were sent up for routine processing.

In addition to booking, PigPass, and *ante mortem* card information, the abattoir records the start and end time that animals in each lot commence being processed. During processing, each carcase receives a unique carcase/body identification number that can be linked to the lot and PigPass information. Therefore, carcases leaving the abattoir can be traced back to the abattoir, to a particular date and time of processing, and to a PigPass consignment and property of origin. Individual carcases are also identified by a:

- Carcase stamp (each carcase/side)
- Carcase tag/ticket attached to one side of each carcase after the scales. This tag identifies:
  - Establishment number,
  - Origin of the stock by means of lot number and vendor code,
  - Body number,
  - Sex,
  - Weight (hot),
  - Fat depth,
  - Slaughter date,
  - Destination of carcase (code),
  - Barcode – to facilitate scanning at point of load-out or boning.

Boning of carcases occurs in batches known as runs. A boning room run may include carcases from multiple lots/sub-lots of pigs processed. Individual carcases are scanned into boning rooms. Boning room scanning systems may preclude pigs not tagged for a specific market from being allowed to enter the boning room. The boning room is a co-mingling point where carcases are processed into smaller cuts. At this point, complete traceability of the meat product back to an individual carcase is lost or becomes fragmented. Typically, meat processed in a boning room can be traced back to a group of carcases using label information, as described below, to determine time of packing, and working backward from there to unique carcase identification numbers that entered the boning room during that time period.

After boning of the carcases, the pork cuts for each run are packed in cartons and bulk bins. In accordance with regulatory requirements, all cartons and bulk bins of product produced in an export-accredited boning room are identified by a label attached to the end panel after weighing. The label identifies, at a minimum:

- Product name (description),
- Product code,
- Inspection stamp (full Australian Inspected for export, or hashed-out Australia Inspected for domestic product),
- Refrigeration statement,
- Country of origin,
- Packed by/ packed for statement,
- Product net weight,
- Packing date,
- Carton number – which is used in conjunction with the product code and packing date to identify the time of packing of the product in question, using the site’s computerised traceability system.

Depending on customer requirement, packed product may also be identified by a brand present on packaging. Between boning runs, all customer-branded packaging is removed from the lines to prevent any misidentification of product for the next production run. If branded packaging is still present at packing stations, they are cleared prior to meat from the next run reaching the stations.

Slaughter dates necessary for export labelling and documentation, as required by importing country requirements, are provided using the site’s computerised traceability system. This is done by selecting the earliest and latest slaughter dates for carcases boned during the boning run in question.

Offal is packed in the offal rooms. All cartons, barrels and bulk bins containing finished offal product are also labelled. The label includes a minimum of:

- Product name (description),
- Product code,
- Inspection stamp (full Australian Inspected for export, or hashed-out Australia Inspected for domestic product),
- Refrigeration statement,
- Country of origin,
- Packed by/ packed for statement,
- Product net weight,
- Packing date,
- Carton number – which is used in conjunction with the product code and packing date to identify the time of packing of the product in question, using the site’s computerised traceability system.

The information on the carcase, carton or bulk bin label is shared by the abattoir with the next physical stakeholder within the material flow of the supply chain as well as the operator. Other stakeholders in the supply chain will not have access to information stored in the private databases of these actors regarding processed meat products, by-products, and co-products. Different abattoirs and their operators also use different software and product label codes which implies limited digital interoperability.

Upon reaching the downstream supply chains, at stakeholders such as value-added processors, wholesalers, butchers, or retailers level, the meat products are typically relabelled. However, the barcodes on the pork products at the wholesalers, retail stores and other downstream actors are linked to the processor barcode. The data about the meat product flow are owned and curated by the owner of the product at the time and where relevant, those processing it, e.g., value-added processors, wholesalers, butchers, and retailers. These data are not registered in a publicly accessible database like PigPass. Thus, there is a fragmented data transmission (supplier-customer based traceability approach) from the point when the carcases or meat product, by-product, co-product leaves the abattoir.

**References**

Australian Bureau of Statistics (2023) Quarterly statistics of livestock slaughtered and meat production. Retrieved from: <https://www.abs.gov.au/statistics/industry/agriculture/livestock-products-australia/latest-release#data-downloads>

Hernández-Jover, M., Schembri, N., Toribio, J.-A., & Holyoake, P. K. (2008) Biosecurity risks associated with current identification practices of producers trading live pigs at livestock sales. *Animal*, *2*(11), 1692–1699. doi: <https://doi.org/10.1017/S1751731108003066>

Hernández-Jover, M., Wu, M., Schembri, N., Holyoake, P. K., & Toribio, J. A. L. M. L. (2008) Evaluation of the official identification system for pigs for sale in New South Wales. *Journal of Animal Science*, *86*(2), 472–475. doi: https://doi.org/[10.2527/jas.2007-0623](https://doi.org/10.2527/jas.2007-0623)

PigBLUP (2022) A PC based genetic evaluation system. Retrieved from: <http://agbu.une.edu.au/pig_genetics/pigblup.html>

Schembri, N., Hart, K., Petersen, R., & Whittington, R. (2006) Assessment of the management practices facilitating the establishment and spread of exotic diseases of pigs in the Sydney region. *Australian Veterinary Journal*, *84*(10), 341–348. doi: <https://doi.org/10.1111/j.1751-0813.2006.00035.x>

Sithole, F., Toribio, J., Schembri, N., & Holyoake, P. K. (2009) Challenges posed to the traceability of weaner pigs following live auction. *Australian Veterinary Journal*, *87*(4), 125–129. doi: <https://doi.org/10.1111/j.1751-0813.2009.00407.x>

ABARES. (2022). *Agricultural commodities and trade data. Agricultural commodities: March quarter 2022 - Statistical tables*. Canberra, Australia: Australian Bureau of Agricultural and Resource Economics and Sciences (ABARES) Retrieved from <https://www.awe.gov.au/abares/research-topics/agricultural-outlook/data#agricultural-commodities>

Australian Bureau of Statistics. (2023). *Quarterly statistics of livestock slaughtered and meat production.* Retrieved from <https://www.abs.gov.au/statistics/industry/agriculture/livestock-products-australia/latest-release#data-downloads>

1. https://www.gumtree.com.au/s-livestock/c18457 [↑](#footnote-ref-1)
2. https://www.australiantenders.com.au/search/tenders/ [↑](#footnote-ref-2)
3. https://pigpass.australianpork.com.au/faq [↑](#footnote-ref-3)
4. Operators can include brokers or agents. These stakeholders are not included in Figure 1 (main article) since these stakeholders do not physically handle material in the supply chain but can take material ownership and make decisions about the material flow. [↑](#footnote-ref-4)
5. Operators, such as brokers or agents, are not shown in Figure 1 as they do not physically handle materials but influence flow decisions. [↑](#footnote-ref-5)
6. Data source: <https://www.abs.gov.au/statistics/industry/agriculture/livestock-and-meat-australia/latest-release> [↑](#footnote-ref-6)
7. http://agbu.une.edu.au/pig_genetics/pigblup.html [↑](#footnote-ref-7)
8. <https://pigpass.australianpork.com.au/faq> [↑](#footnote-ref-8)
